# The Influence of Weather Anomalies on Mercury Cycling in the Marine Coastal Zone of the Southern Baltic—Future Perspective

**DOI:** 10.1007/s11270-014-2248-7

**Published:** 2014-12-11

**Authors:** Magdalena Bełdowska

**Affiliations:** Institute of Oceanography, University of Gdansk, Av. Piłsudskiego 46, 81-378 Gdynia, Poland

**Keywords:** Mercury, Weather anomalies, Climate change, Storms, Coastal zone, Baltic sea

## Abstract

Despite the decreased emission loads of mercury, historical deposits of this metal in various compartments of the environment may become an additional diffuse source in the future. Global climate change manifests itself in the temperate zone in several ways: warmer winters, shorter icing periods, increased precipitation and heightened frequency of extreme events such as strong gales and floods, all of which cause disturbances in the rate and direction of mercury biogeochemical cycling. The present study was conducted at two sites, Oslonino and Gdynia Orlowo (both in the coastal zone of the Gulf of Gdansk), from which samples were collected once a month between January 2012 and December 2012. In the Southern Baltic region, climate changes can certainly enhance coast to basin fluxes of mercury and the transfer of bioavailable forms of this metal to the food web. They may also, in the future, contribute to uncontrollable increases of mercury in the seawater.

## Introduction

Despite the unequivocal usefulness of mercury in many branches of industry, this element is characterised by high toxicity. Indeed, it is the second most toxic heavy metal, after plutonium. So far, no positive functions of mercury have been identified in organisms. On the contrary, as a highly neurotoxic substance, it causes the development of autism in children, while in adults it is responsible for, among other things, Alzheimer’s, Parkinson’s, Lupus and amyotrophic lateral sclerosis (Zahir et al. 2005). Humans have used mercury for thousands of years but, since the early twentieth century, its usage has increased rapidly in many branches of industry. Only in the 1950s, when several hundred of people died after consumption of mercury-enriched fish, did humanity realise how toxic mercury actually is (Ebinghaus et al. 1999). This and similar disasters brought a concerted effort to restrict its usage and decrease its emissions into the environment through various anti-pollution policies.

In recent papers, the influence of climate change on mercury biogeochemical cycles in the Northern region has been widely discussed (Barkay and Poulain 2007; Stern et al. 2012; Douglas et al. 2012). In most areas, mercury emissions into marine or lacustrine environments have been observed to decrease (Korpinen et al. 2010; Pacyna et al. 2010); however, mercury concentration in fish does not decrease proportionally and in some areas even increases (Carrie et al. 2009b; Fjeld and Rognerud 2011). One of the reasons for this may be an enhanced transfer of metals from terrestrial to marine environments, as has been observed in Arctic and subarctic water bodies, where mercury transported through the atmosphere from the northern hemisphere was deposited in peats for decades. Present day climate warming in this area tends to result in the defrosting of peatlands located in permafrost and, together with melt water and mud, historical mercury deposits stored therein are subsequently transported into various water bodies (Barkay and Poulain 2007; Fjeld and Rognerud 2011; Hermanns et al. 2011; Rydberg et al. 2011; Stern et al. 2012; Douglas et al. 2012). Intense rains also contribute to enhanced mercury leaching from river catchments, as was noted in the Mackenzie River (Canada) by Carrie et al. (2009a). When terrestrial mercury enters a water body by such means, the water is also under the influence of climate. For example, it has been proven that algae development and mercury accumulation are higher during warmer and wetter periods than during colder and drier periods (Fjeld and Rognerud 2011; Hermanns et al. 2011; Rydberg et al. 2011). Climate warming also induces increase in plankton biomass, which may result in elevated mercury concentrations in fish (Carrie et al. 2009b), and as was observed in Kusawa Lake (Canada) and some arctic lakes, heightened metal input to sediments (Sanei et al. 2009; Stern et al. 2009). There are a few studies describing the consequences of weather anomalies on the cycles of contaminants (Barkay and Poulain 2007; Douglas et al. 2012; Heimbürger et al. 2010; Stern et al. 2012). However, no studies have so far been published concerning the reemission or remobilisation of mercury (due to climate change) in the southern zone of the Baltic Sea or, for that matter, anywhere else in the temperate zone.

According to HELCOM’s report, mercury input to the Baltic Sea has decreased by 44 % since the 1990s (Korpinen et al. 2010). Additionally, as a result of climate changes in the Polish coastal zone which have increased the frequency of mild winters (i.e., more days warmer than 5 °C and less colder than 0 °C) and rainy summers (Kożuchowski 2009; IMGW PIB Institute of Meteorology and Water Management National Research Institute 2013; HELCOM 2013), mercury load from the region’s main source, fossil fuel combustion, has decreased (Bełdowska et al. 2012). On the other hand, though, a longer ice-free period in the Gulf of Gdansk means a longer biologically active season, and this greatly influences the biogeochemical cycles of contaminants contained therein. Furthermore, a heightened frequency of extreme weather events, such as storms and floods, has contributed to an increased load of chemical substances entering the waters of the coastal zone (IMGW PIB Institute of Meteorology and Water Management National Research Institute 2013; HELCOM 2013). The aim of this study was to observe the tendency of possible shift in mercury cycle under the influence of climate change in the coastal zone of a boreal environment

## Material and Methods

**Fig. 1:**
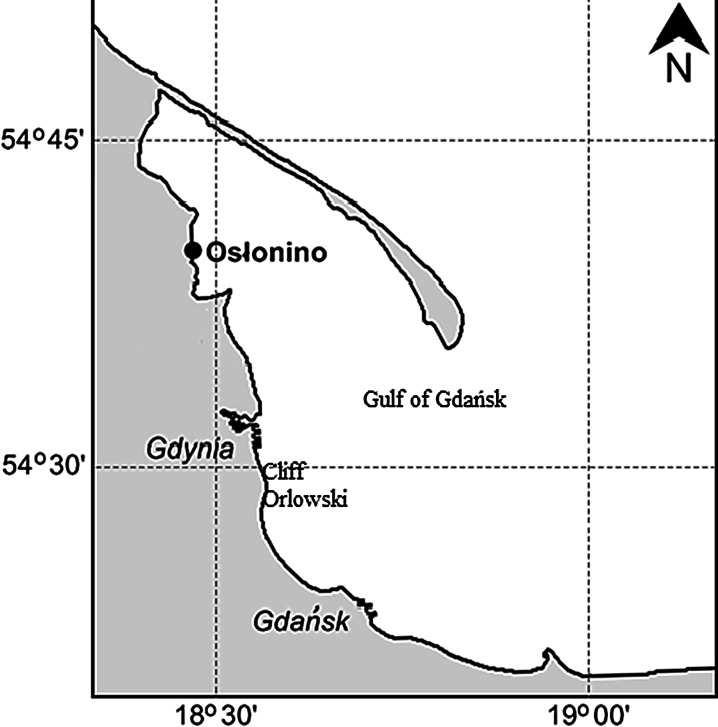
Map of the study area

The study area was situated in Oslonino, in the coastal zone of the Gulf of Gdansk (Southern Baltic) (Fig. 1), where a station was located in a small bay with restricted water exchange. The shallow depth and structure of the bay enhance intensive growth processes in aquatic organisms and this, combined with low water dynamics, results in the accumulation of organic matter. This had a direct influence on the physical and chemical conditions (light and dissolved oxygen) prevailing at the station (Korzeniewski 1993). Samples of phytoplankton, periphyton, sediment and benthic biota were collected once a month from January 2012 to December 2012. Phytoplankton was collected using 20-μm-mesh nets, periphyton was scrubbed from stones, surface sediments were collected by means of a Van Veen Grab (with triple repetition) and benthos samples were obtained by sieving sediment through 0.5 mm mesh.

Additional samples of boulder clay and sand from the cliff at Gdynia Orlowo (Fig. 1) were collected during selected months characterised by intense rain and storms at sea. Seawater samples were also taken from the coastal zone adjacent to the cliff. Over the course of the study period, 5 months fulfilled the conditions mentioned above (intense rain and storms at sea): December 2011, January 2012, March 2012, October 2012 and November 2012. Ten samples of sedimentary material were collected once from different parts of the Orlowo Cliff.

All samples were freeze-dried and homogenised, then analysed using an AMA 254 thermal desorption analyser. The detection limit for solid materials was set at 0.005 ng g^−1^. QA/QC included blank samples, replicates and reference materials: GBW 07314 (sediment) and BCR 279 (Ulva lactuca)—average error did not exceed 5 % (Saniewska et al. 2010a). Seawater samples for mercury analysis were oxidised by adding BrCl and pre-reduced with hydroxylamine hydrochloride solution 1 hour prior to analysis by CVAFS (TEKRAN 2600, Canada), according to US EPA method 1631 (US EPA US Environmental Protection Agency 2002). Quality control procedures for water samples included the use of blanks and water spiked with mercury nitrate within a range of 0.5–25 ng dm^−3^ and produced adequate precision (1 % RSD) and recovery (98–99 %). Each sample was analysed with triple or fivefold repetition and standard deviation was lower than 0.5 ng g^−1^/0.3 ng dm^−3^.

The division into thermally normal and anomal season was excerpted from Climate Monitoring Bulletin issued by the Institute of Meteorology and Water Management–National Research Institute (http://www.imgw.pl/extcont/biuletyn_monitoringu/). This division was made on the basis of data collected during the years 1951–2010 period. Winter 2012 was described as being a thermally normal season, spring and autumn of the same year: anomal warm season and the summer; warm season.

Basing on the meteorological data and information when the heat and power plants starts and ends, two sampling periods were distinguished—heating season (October—end of April) and non-heating season (May—end of September).

Biomass of phytoplankton was determined with an inverted microscope (Nikon TMS, Tokyo, Japan) according to the Utermöhl method (Edler 1979) and HELCOM recommendations (Olenina et al. 2006). Water samples for microscopic analysis of the phytoplankton were preserved using Lugol’s solution (1 %).

Based on the weight of dried phyto, and zoobenthos, and the surface of collected sediment, biomass was then ascertained.

## Results and Discussion

### Hg in the Atmosphere

**Fig. 2:**
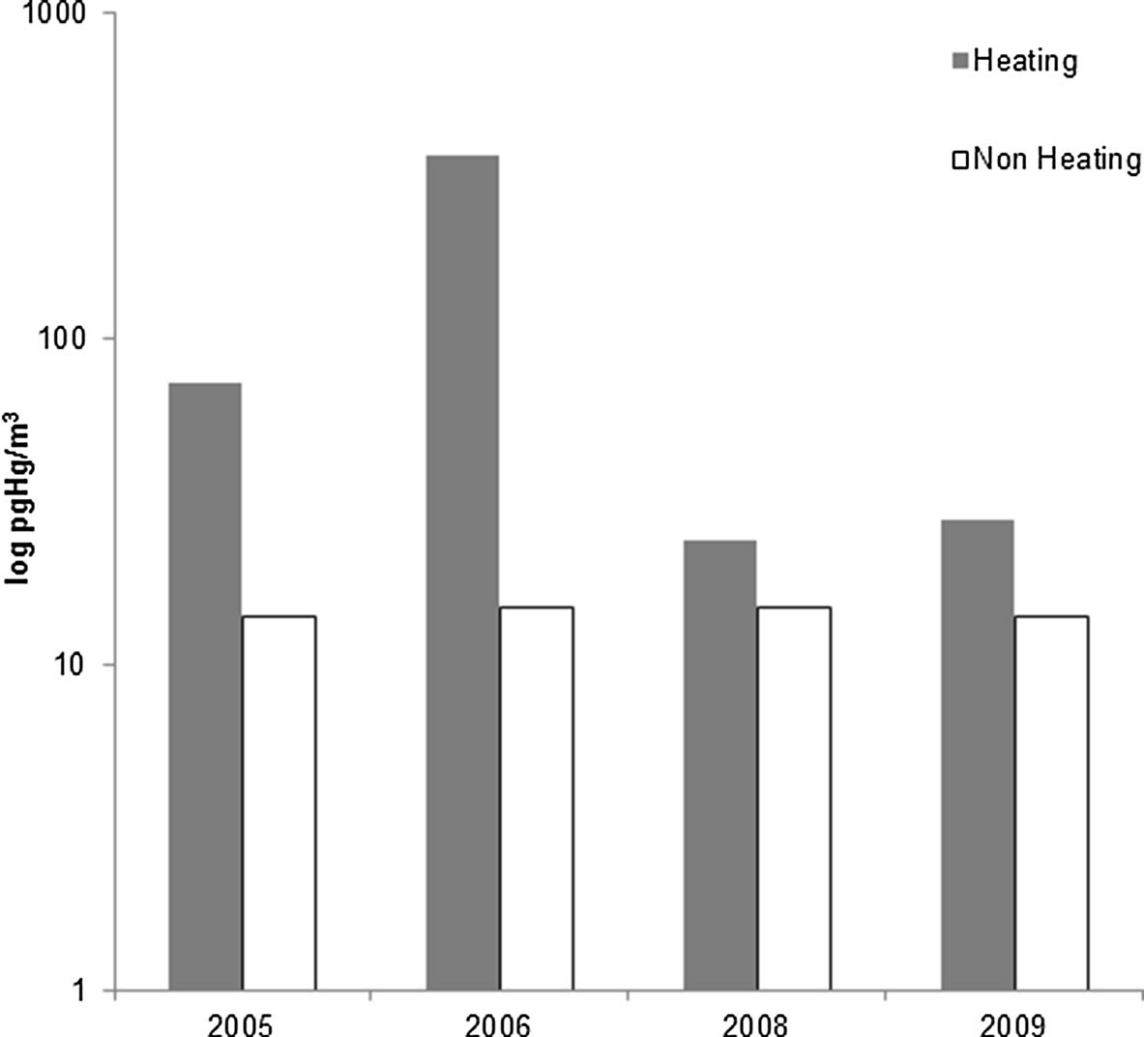
Concentration of Hg in particulate matter in air over Gdynia during the heating and the non-heating season, 2005–2009 (based on Bełdowska et al. 2007, 2012)

Mercury concentration in the air of the coastal zone is governed mostly by the origins of air masses (i.e., maritime or continental) as transformations of gaseous mercury to particulate form favour warm and humid marine air, in which chlorine and bromine ions are abundant (Bełdowska et al. 2014). A mild winter tends to result in less fossil fuel combustion, which is considered to be the major source of mercury in the Southern Baltic (Bełdowska et al. 2007; Bełdowska et al. 2012). In the Gulf of Gdansk area, during the very cold winter of 2006 (average temperature −1.4 °C; minimum −30 °C), average Hg concentrations in the particles in the air equalled 479 pg m^−3^, while in 2008, when the winter was substantially warmer (average temperature 4.8 °C; minimum −1 °C), a 20-fold lower Hg concentration of 21 pg m^−3^ was measured (Bełdowska et al. 2007; Bełdowska et al. 2012) (Fig. 2). The effect of a warm winter (in comparison with a very cold winter) on the mercury cycle, taking into account only dry deposition flux, is an eightfold decrease of annual Hg atmospheric deposition (Bełdowska et al. 2014). Since 1988 (when warm winters began to prevail), the average winter temperature has been reported to exceed the multiyear normal on 15 occasions (IMGW PIB Institute of Meteorology and Water Management National Research Institute 2013).

The influence of climate change can also be seen in the summer season. At this time, in the warm marine air, Hg (0) was most probably oxidised by halogen radicals to form Hg (II) and adsorbed on condensation nuclei, subsequently to be washed out from the atmosphere during rainfall. In months when a higher amount of precipitation occurred, the wet deposition of Hg was higher (Saniewska et al. 2014b). From July to August, more and more anomalies were noted to occur in terms of monthly precipitation totals and, in July 2012, this value reached 220 % of the 1971–2000 normal period (IMGW PIB Institute of Meteorology and Water Management National Research Institute 2013). Assuming that the average concentration of Hg in the rain was comparable to the values measured in 2008 (Saniewska et al. 2014b), the increase in amount of precipitation contributed to several dozen percent increase in annual atmospheric Hg deposition.

### Hg Leaching from Soils

An increasing frequency of extreme weather events has been observed in the Southern Baltic region (HELCOM 2013), leading to increase land erosion (frequently of forest soils, as in the areas of Oslonino, Gdynia Orlowo and Jastrzebia Gora) as a result of both strong winds and increased average wave height. As a consequence, increasing amounts of metals, hitherto stored in terrestrial reservoirs for many years, are reaching the sea in strong, distinct pulses (Bogacka 2003). In freshly eroded peat at Gdynia Orlowo, mercury concentration was found to range from 10 to 60 ng g^−1^ d.w., a concentration which is several dozen times higher than in the coastal sediments. Abrasion of the cliff in Orlowo causes around 47,000 t a^−1^ of the material to be introduced into the Gulf of Gdansk (Bogacka 2003), which translates into about 2.6 kg Hg a^−1^ (Jędruch et al. 2013). Owing to this alone, a large load of mercury enters the coastal zone in a relatively short time, the mass of which is almost two times higher than the total annual amount of mercury transported into the Gulf of Gdansk by the Reda River (the second largest river discharging into the gulf, average flow; 4.3 m^3^ s^−1^) (Saniewska 2013). In the direct aftermath of a storm causing visible cliff erosion here, mercury concentration in the coastal water of the inner part of the Gulf of Gdansk increased from 0.7 ng dm^−3^ before the event to as much as 61.6 ng dm^−3^ after.

In the Gulf of Gdansk area, intensive rainfall and more frequent floods contribute to the remobilisation of labile, land-based mercury species and their transport into the Baltic Sea. Metal leaching is additionally enhanced by low pH rainwater and in the coastal zone of the Baltic Sea, acid rains were found to prevail for the majority of the year (Siudek et al. 2006). More frequent heavy rainfalls also contribute to the leaching of mercury from land, where it has hitherto been deposited for decades. For example, during a extreme flood in 2010 (20 May–19 June 2010), an estimate of the mercury load which was introduced to the Gulf of Gdansk as a direct result was equal to 75 % of the total mercury load for that year (Saniewska et al. 2014a).

### Hg in Seawater

**Fig. 3:**
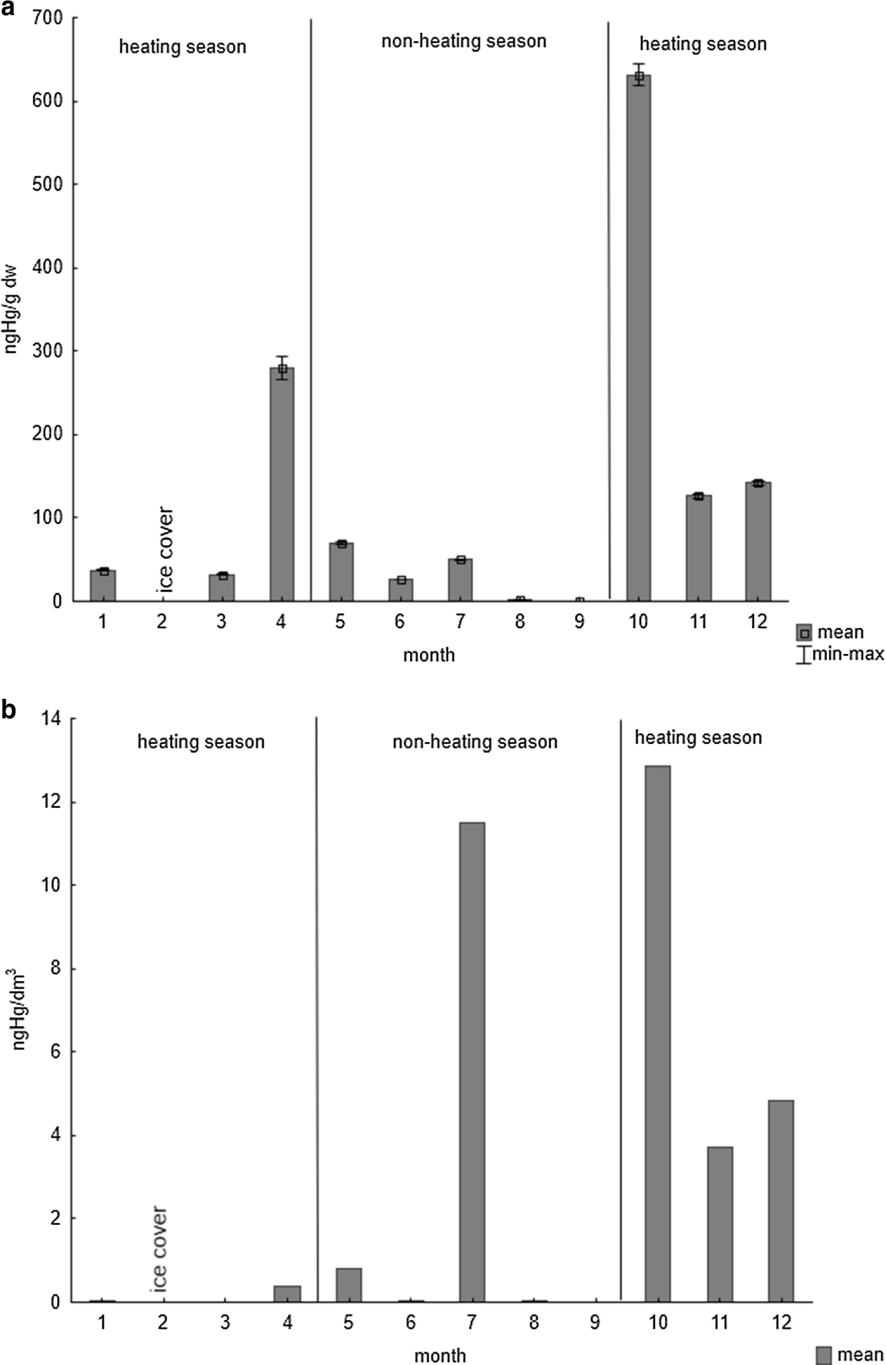
Concentration of Hg: **a** in phytoplankton (ng g^−1^ d.w.); **b** in biomass of phytoplankton (ng dm^−3^) during 2012, in the Gulf of Gdansk

When a thermally normal winter starts later in the year, thereby prolonging the warm season, phytoplankton blooms may be observed in late autumn and even in early calendar winter (Łysiak-Pastuszak et al. 2011). Such was the case in autumn 2012 (phytoplankton was present until December) and, in this situation, mercury entering the coastal zone with atmospheric deposition, the concentration of which is higher at this time of the year (Fig. 2), did not deposit on the bottom of the reservoirs but was accumulated by algae. Consequently, the concentration of mercury determined in phytoplankton during the heating season (especially in autumn) was higher than in the non-heating season (Fig. 3a). Increasing of the Hg concentration in the phytoplankton in late autumn or early spring was also observed during studies conducted at coastal zone of the Gulf of Gdansk in years 2006–2008 (Saniewska et al. 2010b). Elevated metal concentration in phytoplankton and high biomass of them resulted in raised concentration of Hg in the first link of trophic web (Fig. 3b). Any period of extended presence regarding the former can substantially enhance mercury transfer up the food chain of the Baltic Sea per annum.

**Fig. 4:**
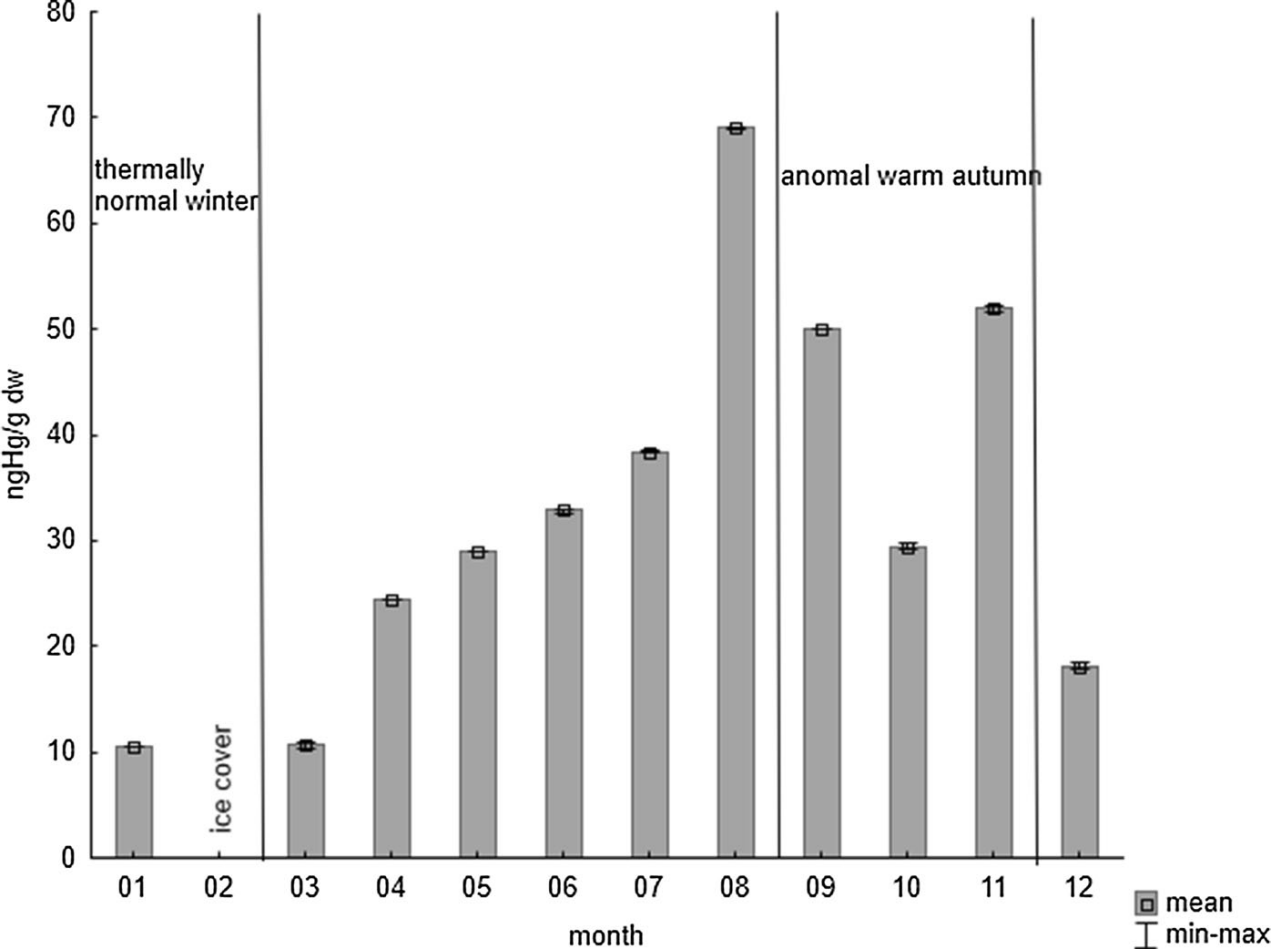
Concentration of Hg in periphyton from stones (ng g^−1^ d.w.) during 2012, in the Gulf of Gdansk

Elevated concentrations of Hg during the thermally anomal warm autumn of 2012 were also observed in the peryphiton gathered from stones (Fig. 4), the average Hg concentration at this time being three times higher than during the thermally normal winter. Therefore, over the course of a warm year, even if Hg input to the southern Baltic Sea is observed to decrease (Bełdowska et al. 2012), the mercury load entering the food web may still exceed that of a year considered to be thermally normal. During the last 10 years, seven autumn seasons were deemed to be warmer than thermally normal (IMGW PIB Institute of Meteorology and Water Management National Research Institute 2013).

### Hg in the Sediments

**Fig. 5:**
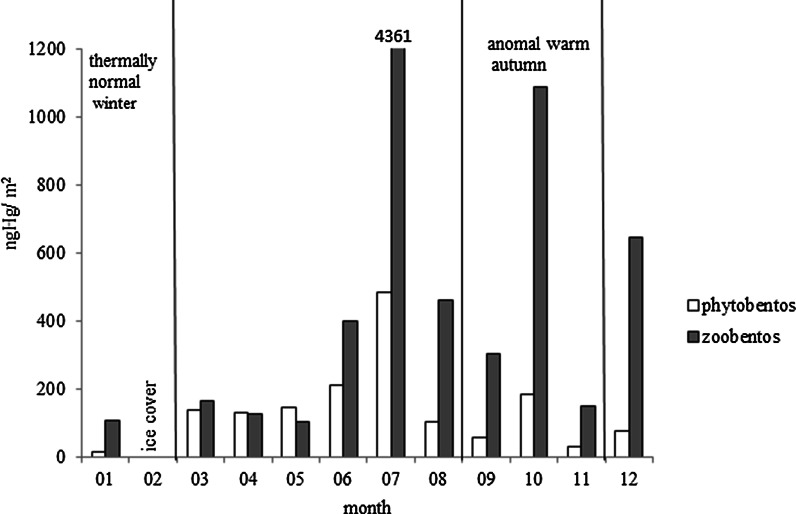
Concentration of Hg in biomass of phytobentos and zoobentos (ng m^−2^) during 2012, in the Gulf of Gdansk

Climate change in the Polish coastal zone manifests itself in the shortening of icing periods (IMGW PIB Institute of Meteorology and Water Management National Research Institute 2013), which affects the fluxes of chemicals in the surface sediments of the coastal zone. In the anomal warm autumn of 2012, an extended presence of phytoplankton was observed and, as a result, an increased amount of dead organic matter (decaying phytoplankton) sank to the bottom of the gulf. Consequently, Hg bound to sedimentary organic particulate matter (Hg_LOI_) was found to be proportional to Hg in suspended particulate matter (Hg_SPM_) (r = 0.75, *p* < 0.05) (Bełdowski et al. 2013). Benthic organisms, also experiencing intensive growth as a result of the warm autumn, were then bioaccumulated the Hg (Fig. 5) and thus a larger mercury load was made available to higher trophic levels. During this period, the concentration of Hg measured in benthos biomass (phyto- and zoobenthos) was five times higher than that of a thermally normal winter. Similarly observations were conducted in previous years (2006–2008): The highest concentration of Hg in phytobenthos was measured during winter without ice cover (Saniewska et al. 2010a).

Sediments, which act as a natural sink for heavy metals in the marine environment, contain historical deposits of metals. In the Baltic Sea, sedimentation rate is quite low (1–2 mm a^−1^) (Bełdowski and Pempkowiak 2009) and mercury deposited during the past 50 years therefore rests in the uppermost 10 cm of its sediments. As this is close to sediment mixing depth, which in this area reaches up to 8 cm below the sediment water interface, it is possible for such mercury deposits to interact with near bottom water. Based on studies conducted in the years 1998–2002, due to a range of biochemical and physical processes, such as organic matter diagenesis, porewater diffusion and resuspension of unconsolidated surface sediments, about 50 % of mercury deposited onto the floor of the Gdansk Basin is reemitted from marine sediments back into the water column (Bełdowski et al. 2009). In this current situation, the equilibria between processes responsible for the formation of stable mercury compounds, and the reemission of mercury back into water column has almost been reached. As a result, if the input of mercury to sediments increases further (with floods, land erosion and dead organic matter), its remobilisation may be expected to increase from several to several dozen percent more than the increase of input. Diffusive fluxes of Hg in the Gulf of Gdansk can reach −5 ng cm^−2^ a^−1^, while fluxes associated with sediment erosion might be as high as −3.3 ng cm^−2^ a^−1^, and this is close to the net accumulation of mercury −5.5 ng cm^−2^ a^−1^ (Bełdowski et al. 2009). Additionally, changes to environmental conditions on the sea bottom (as a result of climate change), such as increased supply of organic matter, decay of massive phytoplankton blooms on the seafloor leading to oxygen deficit and growth of sulfur reducing bacteria, and changes in salinity, may increase the intensity of organic matter diagenesis yet further. It is therefore highly conceivable that, in the future, marine sediments could become a significant and diffuse source of mercury without any reasonable means of control. Consideration of such a scenario is especially important with regards to the Gdansk Basin, where previous research has indicated high contents of labile mercury species in sediments and near-bottom suspended matter (Bełdowski and Pempkowiak 2007; Bełdowska et al. 2013).

## Conclusion

Mercury input from atmosphere, land and marine sediments to the waters of the Southern Baltic coastal zone is highly dependent on changing meteorological parameters. These include variation in seasonal temperature, shortening of the icing period and frequency of extreme weather events and, in the future, such climate changes may result in an uncontrollable increase of bioavailable mercury in the seawater. An anomal or extremely warm winter, such as was experienced in the Southern Baltic in 2008, undoubtedly contributes to a significant reduction in Hg dry deposition fluxes. At the same time, however, Hg entering seawater is retained in the trophic chain to a larger extent than it would be during a thermally normal winter. Lack of icing, plankton blooms and benthic development in late autumn or even winter, maintain Hg bioavailability for an extended time period in comparison with that of a thermally normal season. In addition, benthic organisms contribute to the remobilisation of Hg from sediments where it has been deposited for many years. Floods and storms also contribute to the remobilisation of metals, generally from historical land-based deposits. Such processes are of major significance to marine organisms inhabiting the coastal zone and estuaries, as they cause toxic substances to be introduced into the sea within a relatively short time. The results presented as part of this study indicate that climate change can indeed enhance mercury coast to basin fluxes, as well as the transfer of bioavailable forms of this metal into the food web; however, they do not allow for proper quantification of those processes. Future studies concerned with mercury behavior in the temperate zone under influence of climate change need to be extended to plankton and benthos species composition, preferably during periods which feature abnormally high temperatures in autumn, winter and spring. It is therefore intended that further connected research, which is planned for 2012–2014 in the area of the Gdansk Basin and which will be conducted within the framework of a National Science Centre (NSC) project, will both address these requirements and provide some quantification of observed fluxes.
